# Editorial: Is insulin resistance the Eminence Grise of aging and non-communicable chronic diseases?

**DOI:** 10.3389/fendo.2025.1735592

**Published:** 2025-11-13

**Authors:** Dzilda Velickiene, Izabela Szymczak-Pajor, Aivaras Ratkevicius

**Affiliations:** 1 Institute of Endocrinology, Lithuanian University of Health Sciences, Kaunas, Lithuania; 2 Department of Nucleic Acid Biochemistry, Medical University of Lodz, Łódź, Poland; 3 Queen Mary University of London, London, United Kingdom

**Keywords:** insulin resistance, non-communicable chronic diseases, aging, cardiovascular diseases, non-insulin-based surrogate indices for insulin resistance

Insulin resistance (IR) is recognized as a central mechanism in metabolic dysfunction, with its influence extending far beyond glucose regulation. Increasingly, IR is recognized as the Eminence Grise — the unseen but powerful driver — behind many non-communicable chronic diseases (NCDs) and the biological processes of aging. The Research Topic “Is insulin resistance the Eminence Grise of aging and NCDs?” brings together 12 original investigations and reviews that collectively expand our understanding of IR as a systemic, multi-organ phenomenon. These studies explore IR not only as a metabolic hallmark but as a unifying pathophysiological thread linking cardiovascular, renal, hepatic, respiratory, neurological, and psychological disorders across the lifespan. The reviewed studies explored various accessible, non–insulin-based surrogate indices [Triglyceride-Glucose (TyG), Estimated Glucose Disposal Rate (eGDR)] across aging-related diseases. Although the hyperinsulinemic–euglycemic clamp remains the most accurate method for assessing IR, its application is often limited by the complexity and constraints of clinical settings.

## Current knowledge and gaps

Decades of research have established that IR contributes to a wide range of metabolic and degenerative diseases. It plays a fundamental role in the development of type 2 diabetes, dyslipidemia, non-alcoholic fatty liver disease (NAFLD), and atherosclerosis (1, 2). Mechanistically, IR arises from the interplay between genetic susceptibility, ectopic lipid accumulation, mitochondrial dysfunction, chronic inflammation, and altered adipokine signaling (3). Beyond classical metabolic organs, impaired insulin signaling affects endothelial cells, neurons, and immune responses, contributing to vascular stiffness, neurodegeneration, and systemic low-grade inflammation — hallmarks of aging (1–5).

However, despite this well-established framework, several aspects remain underexplored. While the molecular basis of IR has been elucidated in skeletal muscle, liver, and adipose tissue, much less is known about its role in non-traditional target organs such as the lungs, kidneys, or brain. Moreover, there remains a gap in understanding the predictive and diagnostic value of emerging non–insulin-based IR indices, their relevance in acute settings, and their relationship with mental health and cognitive decline. Gaps also persist regarding pharmacological modulation, adipose–immune crosstalk, and longitudinal mechanistic studies integrating omics and imaging biomarkers.

The papers in this Topic address several of these gaps, validate surrogate markers, and deepen our understanding of IR.

## IR and cardiovascular diseases

The interplay between IR and cardiovascular disease (CVD) remains a subject of persistent inquiry. Zhang et al. studied patients with T2DM suffering acute myocardial infarction and identified a strong correlation between the TyG index — a surrogate of IR — and major CVD. Their findings underscore TyG’s potential as a valuable prognostic tool in this vulnerable population, highlighting how metabolic derangements aggravate cardiovascular risk even when left ventricular systolic function is preserved.

Complementing these findings, Wang et al. assessed hyperuricemia risk through eGDR, another non–insulin-based IR measure. Their results reinforce the notion that systemic metabolic inefficiency, reflected by lower eGDR, predisposes to urate accumulation — further linking IR to vascular and renal injury pathways.

## IR role in acute and chronic renal disorders

Using the MIMIC-IV database, Wang et al. demonstrated that elevated TyG–body mass index is associated with both acute kidney injury and the need for renal replacement therapy in critically ill septic patients. These results extend the clinical significance of IR markers into acute care settings, showing their utility in identifying patients at higher risk of renal deterioration.

Zhang et al. analyzed associations between non–insulin-based IR indices and chronic diabetic nephropathy in U.S. adults (NHANES data). They confirmed that higher IR indices correspond to a greater prevalence of nephropathy, supporting their use for early detection of renal complications in diabetes care.

## IR and liver diseases

Cao et al. revealed a U-shaped association between the TyG index and incident diabetes among adults with metabolic dysfunction–associated steatotic liver disease. This relationship suggests that both excessively low and high TyG values may be deleterious, reflecting the delicate balance between metabolic flexibility and dysfunction in the liver and underscoring the need for risk stratification in this population. In parallel, Zhao et al. explored the relationship between the single-point insulin sensitivity estimator (SPISE) and NAFLD in individuals with T2DM, demonstrating an inverse correlation and supporting SPISE as a non-invasive marker for hepatic insulin sensitivity in clinical practice.

## IR, metabolic syndrome and population health

The manifestation of IR in clinical populations is strongly mediated by environmental and lifestyle interactions, emphasizing its multifactorial etiology. In a nested case–control study Rong et al. identified demographic, lifestyle, biochemical factors associated with metabolic syndrome among adult, reaffirming the multifactorial roots of IR that intertwine genetic susceptibility, environmental exposure, and behavioral risk.

Expanding to the adolescent population, Villasis-Keever et al. investigated the relationship between anxiety and cardiometabolic risk factors in obese youth using propensity score methods. Their findings highlight that psychological distress and metabolic dysregulation may reinforce each other early in life — positioning IR as a critical link between mental and metabolic health.

## IR and pulmonary structural changes

Emerging evidence suggests that IR manifests in diverse organ systems through complex cellular mechanisms. Lin et al. examined the association between eGDR and preserved ratio impaired spirometry (PRISm), a condition reflecting early restrictive lung dysfunction. Their study revealed that reduced eGDR — a marker of heightened IR — correlates with PRISm, suggesting that systemic metabolic impairment may contribute to pulmonary structural or microvascular changes.

## IR and cognition, brain structure, aging

The influence of IR on the brain is gaining prominence in aging research. Two articles here provide evidence of IR’s role in cognitive decline. Wang et al. evaluated the link between IR and cognitive impairment using the eGDR in a non-diabetic aging population (CHARLS data), demonstrating that IR is an independent risk factor for reduced cognitive function of adults. This finding underscores the systemic impact of IR on neural function, even in the absence of overt diabetes.

Adding a genetic and neuroimaging dimension, Huang et al. used a Mendelian randomization approach to reveal that genetically predicted brain cortical structure mediates the causality between IR and cognitive impairment, providing compelling genetic evidence for a structural link between IR and neurological health. Collectively, these studies reinforce the hypothesis that IR serves as a shared etiological substrate for both metabolic and neurodegenerative diseases — a defining feature of aging biology.

## Reversing the tide: exercise and insulin sensitivity

Lifestyle interventions remain the cornerstone of IR management (5). In their systematic review and network meta-analysis, Pan et al. compared nine distinct exercise modalities and found heterogeneous effects on insulin sensitivity among individuals with diabetes. Aerobic, resistance, and combined training showed the most consistent benefits, but emerging modalities such as high-intensity interval training and mind–body exercises also demonstrated promise. This comprehensive synthesis not only supports personalized exercise prescriptions but also reaffirms the modifiability of IR — even in advanced disease stages.

In conclusion, this Research Topic decisively confirms that IR is the fundamental, hidden mechanism underlying the widespread convergence of aging and NCDs. The utility of validated, accessible indices allows for early and precise risk stratification across varies systems. The collective evidence strongly supports a paradigm shift where future therapeutic research focuses not just on downstream disease management, but on personalized strategies to restore insulin sensitivity and interrupt the devastating cascade initiated by the Eminence Grise. Bringing the hidden influence of IR into the light is the first critical step toward mitigating the global burden of NCDs.

## References

[1] James DE Stöckli J Birnbaum MJ . The aetiology and molecular landscape of insulin resistance. Nat Rev Mol Cell Biol. (2021) 22:751–71. doi: 10.1038/s41580-021-00390-6, PMID: 34285405

[2] Samuel VT Shulman GI . The pathogenesis of insulin resistance: integrating signaling pathways and substrate flux. Cell. (2016) 164:716–29. doi: 10.1172/JCI77812, PMID: 26727229 PMC4701542

[3] Li M Chi X Wang Y Setrerrahmane S Xie W Xu H . Trends in insulin resistance: insights into mechanisms and therapeutic strategy. Sig Transduct Target Ther. (2022) 7. doi: 10.1038/s41392-022-01073-0, PMID: 35794109 PMC9259665

[4] Kahn SE Hull RL Utzschneider KM . Mechanisms linking obesity to insulin resistance and type 2 diabetes. Nature. (2019) 575:85–9. doi: 10.1038/nature05482, PMID: 17167471

[5] Kolb H Kempf K and Martin S . Insulin and aging – a disappointing relationship. Front Endocrinol. (2023) 14:1261298. doi: 10.3389/fendo.2023.1261298, PMID: 37854186 PMC10579801

